# Procyanidin B2 Alleviates Heat-Induced Oxidative Stress through the Nrf2 Pathway in Bovine Mammary Epithelial Cells

**DOI:** 10.3390/ijms23147769

**Published:** 2022-07-14

**Authors:** Hongzhuang Wang, Weiguang Hao, Liang Yang, Tingting Li, Chongchong Zhao, Peishi Yan, Shengjuan Wei

**Affiliations:** College of Animal Science and Technology, Nanjing Agricultural University, No. 1 Weigang Road, Nanjing 210095, China; wanghz20212@163.com (H.W.); haowg21@163.com (W.H.); liang55yang@163.com (L.Y.); tgzy2014@163.com (T.L.); zhaoch0517@163.com (C.Z.); yanps@njau.edu.cn (P.Y.)

**Keywords:** heat stress, mammary epithelial cells, procyanidin B2, Nrf2

## Abstract

The objective of this study was to investigate the protective effects and potential molecular mechanisms of procyanidin B2 (PB2) in MAC-T (mammary alveolar cells-large T antigen) cells during heat stress (HS). The MAC-T cells were divided into three treatment groups: control (37 °C), HS (42 °C), and PB2 + HS (42 °C). Compared with MAC-T cells that were consistently cultured at 37 °C, acute HS treatment remarkably decreased cell viability, reduced activities of catalase (CAT), superoxide dismutase (SOD), and total antioxidant capacity (T-AOC), and elevated intracellular levels of malondialdehyde (MDA) and reactive oxygen species (ROS). Additionally, nuclear factor erythroid 2-related factor 2 (Nrf2) was activated and translocated to the nucleus, in accompaniment with upregulation of Nrf2, heme oxygenase 1 (HO-1), thioredoxin reductase 1 (Txnrd1), and heat shock protein 70 (HSP70). In parallel, both mRNA transcript and actual protein secretion of pro-inflammatory cytokines, including tumor necrosis factor-α (TNF-α) and interleukin-1β (IL-1β), were increased by heat stress. Pretreatment of MAC-T cells with 0~25 μM PB2 alleviated the decline of cell viability by HS in a dose-dependent fashion and protected cells against HS-induced oxidative stress, as evidenced by significantly improved CAT, SOD, and T-AOC activity, as well as with decreased MDA and ROS generation. Furthermore, PB2 further activated the Nrf2 signaling pathway and reversed the inflammatory response induced by HS. Silencing of Nrf2 by si-Nrf2 transfection not only exacerbated HS-induced cell death and provoked oxidative stress and the inflammatory response, but also greatly abolished the cytoprotective effects under HS of PB2. In summary, PB2 protected MAC-T cells against HS-induced cell death, oxidative stress, and inflammatory response, partially by operating at the Nrf2 signal pathway.

## 1. Introduction

Hot temperature is not conducive to the thermal dissipation of dairy cows; it is easy to cause an increase in core body temperature, which, finally, leads to heat stress (HS). This effect is exacerbated in high-yielding dairy cows, due to higher consumption and production thermogenesis. Many negative effects of HS on dairy cows have been reported, including the upregulated oxidative stress status [1], increased susceptibility to mastitis [2], and decreased milk production and quality [3]. Exploring effective ways to attenuate the adverse effects of HS on dairy cows deserves great importance.

During HS, the excessive production and accumulation of cellular reactive oxygen species (ROS) in the mammary epithelia cells of cows leads to oxidative stress injury, thus disrupting cell function and causing the reduction of mammary epithelial cells [4]. Nuclear factor erythroid 2-related factor 2 (Nrf2) is an important transcription factor that regulates the cellular defense against oxidative stress [5]. It has been reported that the activated Nrf2 translocation from the cytosol into the nucleus initiates the gene transcription of several key downstream antioxidant molecules, such as heme oxygenase-1 (HO-1) and thioredoxin reductase-1 (Txnrd1), thus scavenging free radicals and resisting oxidative stress [6]. The Nrf2/HO-1 signaling pathway plays a vital role in regulating intracellular redox by protecting cells from ROS [3]. Additionally, the reduction of ROS via activating Nrf2 can also inhibit the activation of the NF-κB signaling pathway, thereby decreasing the expression of intracellular pro-inflammatory factors [7]. So far, in vivo and in vitro studies have indicated that providing antioxidants can restore the redox balance of cells, enhance the expression of antioxidant enzymes, and improve the antioxidant capacity of dairy cows, which is an effective means to protect dairy cows from HS-induced damage [8,9,10].

Procyanidins, a subclass of a natural polyphenol compound, are widely distributed in plants, including fruits, vegetables, and nuts, etc. [11]. Among various isomers of procyanidins, procyanidin B2 (PB2) is the main dimer with the most extensive source and very powerful antioxidant activity [12]. In addition, PB2 is relatively stable under digestion conditions and detectable in human and rat plasma after dietary consumption [13,14]. Previous studies have revealed that PB2 possesses a variety of bioactivities, including the significant anti-oxidation, anti-inflammation, anti-toxicity, and anti-tumour effects [15,16,17]. However, as a strong antioxidant, little information is available about the protective effects of PB2 in dairy cows under heat exposure, and the underlying molecular mechanism remains to be elucidated. Therefore, we took advantage of HS model of MAC-T (a mammary epithelia cell line) cells to explore the effects of PB2 on HS-induced MAC-T cell damage and clarify the role of Nrf2 signaling pathway in this process.

## 2. Results

### 2.1. PB2 Ameliorates Acute HS-Induced Cell Viability Loss

**Figure 1:**
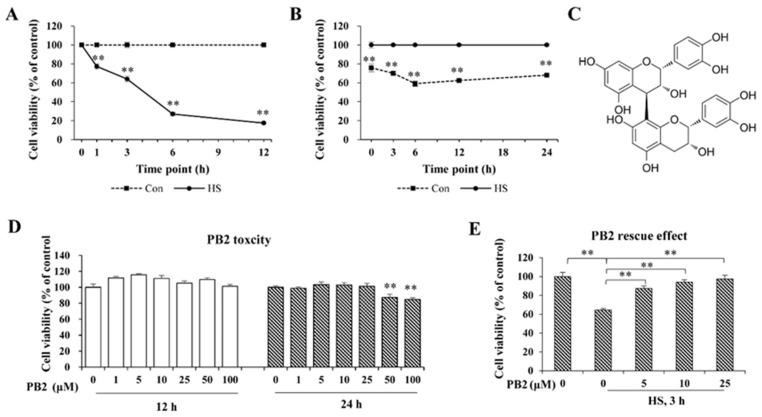
Effect of HS and PB2 on MAC-T cell viability. (**A**) Cell viability was measured in MAC-T cells that were treated with HS (42 °C for the indicated time periods) and then recovered at 37 °C for 6 h. (**B**) Cell viability was measured in cells treated with HS at 42 °C for 3 h and then recovered at 37 °C for the indicated time periods. (**C**) Chemical structure of PB2. (**D**) Cell viability was measured in cells treated with indicated concentrations of PB2 for 12 or 24 h. (**E**) Cells were pretreated with the indicated concentrations of PB2 for 24 h, followed by HS treatment at 42 °C for 3 h and recovery at 37 °C for 6 h. Then, cell viability was determined. *n* ≥ 5. ** *p* < 0.01.

To establish an acute HS model, cells were treated with HS (42 °C) for 1, 3, 6, and 12 h, respectively, and recovered (37 °C) for 6 h; then, cell viability was assessed. As shown in Figure 1A, cell viability sharply decreased at all indicated treatment times (*p* < 0.01) and decreased with the time extension. Furthermore, for cells treated with HS (42 °C) for 3 h and then recovered (37 °C) for 0, 3, 6, 12, and 24 h, the cell viability rate significantly reduced during the whole 0~24 h recovery time period after HS treatment (*p* < 0.01; Figure 1B). Specifically, cell viability markedly decreased with 0 h of recovery time, arrived at the lowest at the 6-h period (59% of control), and gradually increased with the 6–24 h recovery time period. Thus, treatment of HS at 42 °C for 3 h and recovery at 37 °C for 6 h (around 50% cell viability reduction) was used to establish the acute HS model in all of the following tests.

The chemical structure of PB2 is shown in Figure 1C. Co-incubation with various concentrations (1, 5, 10, 25, 50, and 100 μM) of PB2 for 12 h and co-incubation with low concentrations (1, 5, 10, and 25 μM) of PB2 for 24 h did not change cell viability (*p* > 0.05). However, treatment with 50 and 100 μM PB2 significantly reduced cell viability (*p* < 0.01; Figure 1D). Thus, ≤25 μM PB2 for 24 h pretreatment was used in this experiment to detect its protective effect on HS-induced damage in MAC-T cells. As shown in Figure 1E, HS treatment sharply decreased cell viability (*p* < 0.01). Additionally, pretreatment of 5, 10, and 25 μM PB2 significantly alleviated cell viability loss induced by HS, and the protective effect was in a dose-dependent manner.

### 2.2. PB2 Alleviates Oxidative Stress Induced by HS

**Figure 2:**
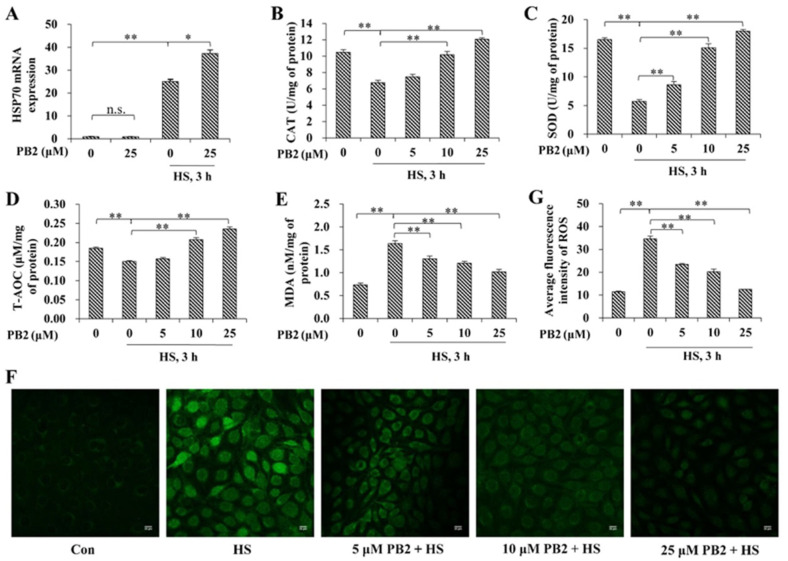
Effect of PB2 and HS on oxidative stress of MAC-T cells. (**A**) MAC-T cells were pre-incubated with or without 25 μM PB2 for 24 h, followed by HS treatment at 42 °C for 3 h and recovery at 37 °C for 6 h. Then, cells were collected to detect *HSP70* mRNA expressions (*n* = 3). Additionally, MAC-T cells were pre-incubated with indicated concentrations of PB2 for 24 h, followed by HS treatment at 42 °C for 3 h and recovery at 37 °C for 6 h. Then, the cells were analyzed for: (**B**) the catalase (CAT) activity; (**C**) the superoxide dismutase (SOD) activity; (**D**) the total antioxidant capacity (T-AOC) activity; and (**E**) the malondialdehyde (MDA) content. (**F**) Images of the MAC-T cells stained with DCFH-DA were captured by a laser scanning confocal microscope, followed by relative florescence intensity assay (**G**). *n* ≥ 6. Scale bar: 50 μm. * *p* < 0.05, ** *p* < 0.01, n.s. *p* > 0.05.

To assess cellular response after heat exposure, mRNA expression of *HSP70* was detected (Figure 2A). Results indicate that 25 μM PB2 did not cause a significant change, concerning *HSP70* mRNA expression (*p* > 0.05), while HS treatment greatly enhanced *HSP70* expression (*p* < 0.01). For cells treated with HS, pre-incubation of 25 μM PB2 further increased *HSP70* expression (*p* < 0.05).

Then, the effect of PB2 and HS on oxidative stress of MAC-T cells were measured. The results (Figure 2B–E) show HS treatment significantly decreased intracellular CAT, SOD, and T-AOC activity and increased MDA content (*p* < 0.01). Pretreatment of 10 and 25 μM PB2 significantly alleviated HS-induced changes of all the above indicated markers (*p* < 0.01), while pretreatment of 5 μM PB2 only played a role in resisting HS-induced SOD and MDA activity change (*p* < 0.01). Preconditioning with 5 μM PB2 failed to relieve CAT and T-AOC activity under HS (*p* > 0.05). ROS contents were visualized via confocal microscopy and quantified via ImageJ. As shown in Figure 2F,G, HS treatment significantly increased intracellular ROS accumulation; pretreatments of 5, 10, and 25 μM PB2 dose-dependently decreased the ROS contents induced by HS (*p* < 0.01).

### 2.3. PB2 Protects against HS-Induced Inflammatory Response

**Figure 3:**
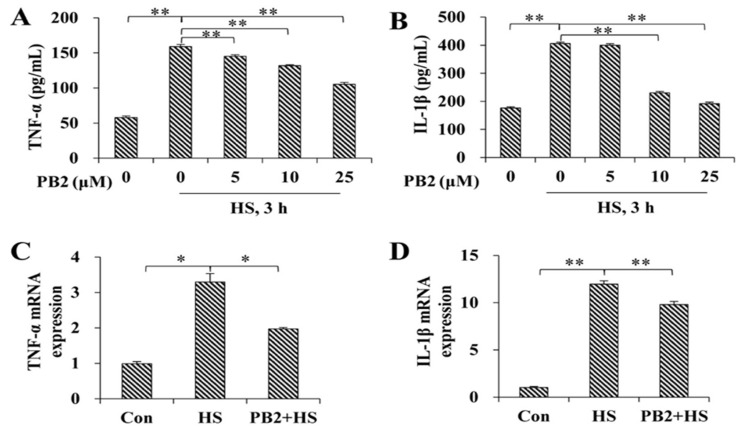
Effect of PB2 and HS on gene expression and release of pro-inflammatory cytokines. MAC-T cells were pre-incubated with different concentrations (0, 5, 10, and 25 μM) of PB2 for 24 h, followed by HS treatment at 42 °C for 3 h and recovery at 37 °C for 6 h. Then, release of (**A**) TNF-α and (**B**) IL-1β in culture media were investigated by ELISA kit. Additionally, after HS and recovery treatment, cells pre-incubated with or without 25 μM PB2 were collected to detect mRNA expressions of (**C**) *TNF-α* and (**D**) *IL-1β*. *n* = 3. * *p* < 0.05, ** *p* < 0.01.

As shown in Figure 3A,B, the release of pro-inflammatory cytokines, including TNF-α and IL-1β, were increased after HS treatment (*p* < 0.01); pretreatment with 5, 10, and 25 μM PB2 significantly decreased their release in a dose-dependent manner (*p* < 0.01), except 5 μM PB2 pretreatment on IL-1β (*p* > 0.05). Consistently, mRNA expressions of *TNF-α* (*p* < 0.05) and *IL-1β* (*p* < 0.01) were enhanced by HS treatment; pretreatment with 25 μM PB2 significantly alleviated the mRNA expressions of *TNF-α* (*p* < 0.05) and *IL-1β* (*p* < 0.01; Figure 3C,D).

### 2.4. PB2 Further Promotes the Activation of Nrf2/HO-1 Signaling Pathway

**Figure 4:**
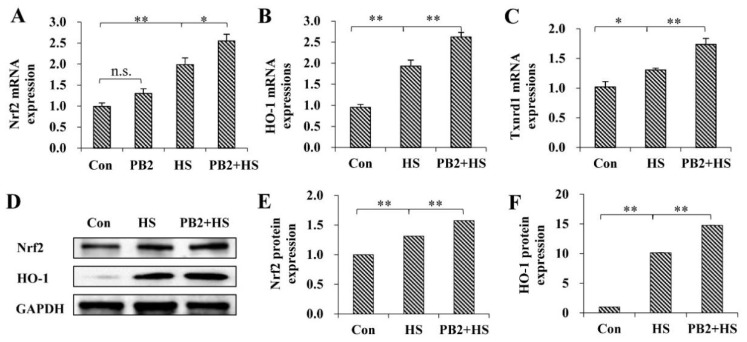
Effect of PB2 and HS on Nrf2 pathway activation of MAC-T cells. MAC-T cells were pretreated with or without 25 μM PB2 for 24 h, followed by HS treatment for 3 h, and then recovered for another 6 h. Then, cells were collected for mRNA and protein expression test. (**A**) *Nrf2* mRNA level. (**B**) *HO-1* mRNA level. (**C**) *Txnrd1* mRNA level. (**D**–**F**) Protein expressions of Nrf2 and HO-1. *n* = 3. * *p* < 0.05, ** *p* < 0.01, n.s. *p* > 0.05.

The activation of Nrf2/HO-1 signaling was investigated. As shown in Figure 4A–C, 25 μM PB2 did not change *Nrf2* mRNA expression (*p* > 0.05), while HS significantly up-regulated *Nrf2* (*p* < 0.01), *HO-1* (*p* < 0.01), and *Txnrd1*(*p* < 0.05) mRNA expressions, and 25 μM PB2 pretreatment further increased mRNA expressions of the *Nrf2* (*p* < 0.05), HO-1 (*p* < 0.01), and Txnrd1 (*p* < 0.01) in MAC-T cells. Consistently, the protein expressions of Nrf2 and HO-1 were enhanced by HS treatment (Nrf2, *p* < 0.01; HO-1, *p* < 0.05), and they further increased by 25 μM PB2 pretreatment (Nrf2, *p* < 0.01; HO-1, *p* < 0.05; Figure 4D–F).

**Figure 5:**
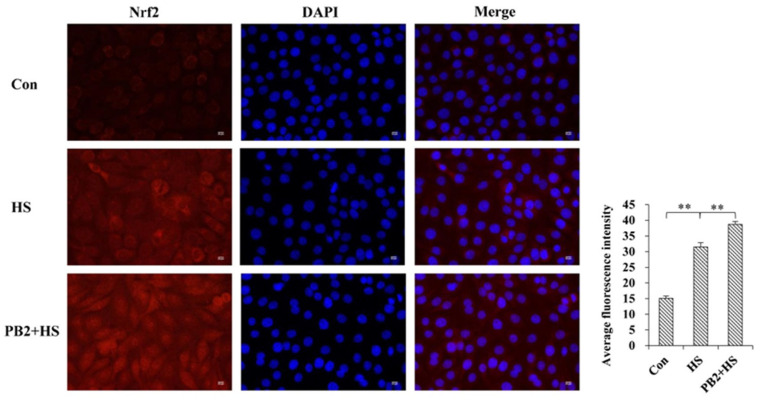
Immunofluorescence confocal microscopy analysis of Nrf2 on its nuclear localization in MAC-T cells. MAC-T cells were pretreated with or without 25 μM PB2 for 24 h, followed by HS treatment for 3 h and recovery for 6 h. Then, cells were stained with anti-Nrf2 antibody (red) and nuclei with DAPI (blue). Experiments were performed three times and representative immunofluorescence images are shown. Relative florescence intensity assay was performed to determine colocalization of Nrf2 with the nucleus. Normalized values are plotted. *n* = 3. Scale bar: 50 μm. ** *p* < 0.01.

In addition, immunofluorescence confocal microscopy analysis show HS treatment stimulated Nrf2 nuclear translocation in MAC-T cells (*p* < 0.01), and 25 μM PB2 pretreatment further promoted the nuclear translocation of Nrf2 (*p* < 0.01; Figure 5).

### 2.5. Nrf2 Knockdown and Its Effect on MAC-T Cell Viability

**Figure 6:**
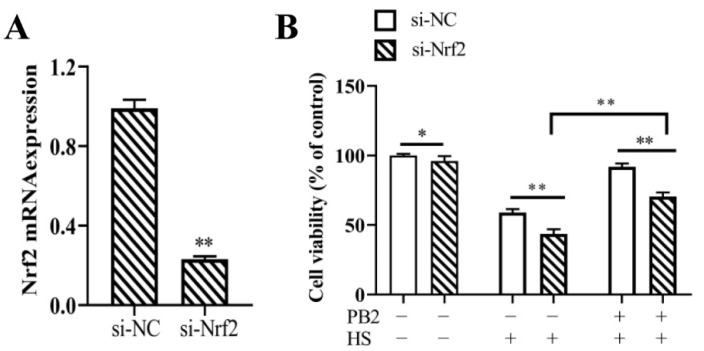
Efficiency of Nrf2 knockdown and its effect on MAC-T cell viability. (**A**) MAC-T cells were transfected with si-Nrf2 or si-NC for 12 h. After 48 h of transfection, cells were collected to detect Nrf2 mRNA expression level. *n* = 3. (**B**) Additionally, MAC-T cells were transfected with si-Nrf2 or si-NC for 12 h, then incubated with/without 25 μM PB2 for 24 h, followed by HS treatment for 3 h and recovery for 6 h. Then, cell viability level was determined. *n* = 6. * *p* < 0.05, ** *p* < 0.01.

In order to explore functions of the Nrf2 signaling pathway in PB2 protective effects on HS-induced cell damage, siRNA-mediated *Nrf2* gene silencing was carried out in the following experiments. As shown in Figure 6A, compared with the control (si-NC) group, transfection of si-Nrf2 significantly reduced 76% of *Nrf2* mRNA levels (*p* < 0.01), indicating si-Nrf2 could be used in this experiment. Compared with the si-NC group, the transfection of si-Nrf2 significantly reduced cell viability under the normal culture condition of 37 °C (*p* < 0.05), the 42 °C HS condition (*p* < 0.01), as well as the PB2 + HS condition (*p* < 0.01; Figure 6B). However, comparing with si-Nrf2 + HS groups, 25 μM PB2 pretreatment still significantly enhanced cell viability (*p* < 0.01). These results suggest Nrf2 played a mild negative role on cell viability, and the PB2 protective effect on HS-induced cell viability loss was partly through the Nrf2 signaling pathway.

### 2.6. PB2 Alleviates HS-Induced Oxidative Stress and Inflammatory Response through the Nrf2 Signaling Pathway

**Figure 7:**
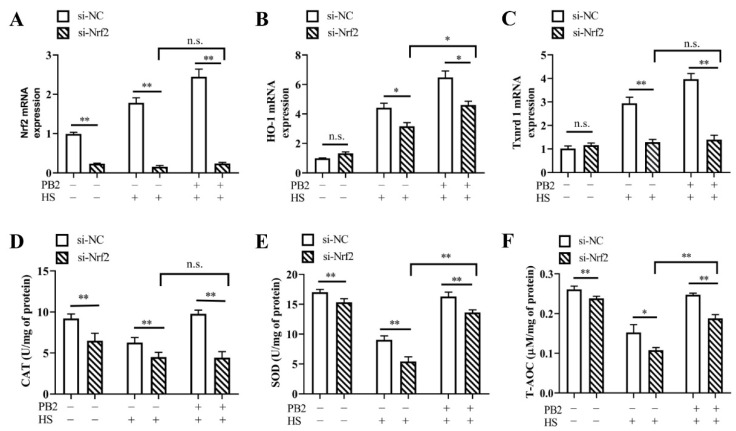
Effect of Nrf2 knockdown on oxidative stress in MAC-T cells. MAC-T cells were transfected with si-Nrf2 or si-NC for 12 h, and then incubated with/without 25 μM PB2 for 24 h, followed by HS treatment for 3 h and recovery for 6 h. Then, the cells were analyzed for (**A**) *Nrf2* mRNA, (**B**) *HO-1* mRNA, and (**C**) *Txnrd1* mRNA levels. *n* = 3. Additionally, (**D**) the catalase (CAT) activity, (**E**) the superoxide dismutase (SOD) activity, and (**F**) the total antioxidant capacity (T-AOC) activity were detected. *n* ≥ 6. * *p* < 0.05, ** *p* < 0.01, n.s. *p* > 0.05.

Effect of Nrf2 knockdown on HS-induced oxidative stress in MAC-T cells was detected. Results in Figure 7A–C show Nrf2 silencing remarkably decreased *Nrf2* mRNA levels (*p* < 0.01), with mRNA levels of *HO-1* and *Txnrd1* unchanged (*p* > 0.05). Under both HS and PB2 + HS conditions, Nrf2 knockdown significantly decreased mRNA expressions of *Nrf2* (*p* < 0.01), *HO-1* (*p* < 0.05), and *Txnrd1* (*p* < 0.01). Furthermore, compared to the si-Nrf2 + HS groups, 25 μM PB2 pretreatment did not change the *Nrf2* and *Txnrd1* mRNA levels (*p* > 0.05), with *HO-1* mRNA levels enhanced (*p* < 0.05). The activities of CAT, SOD, and T-AOC reveal a similar effect of Nrf2. As shown in Figure 7D–F, Nrf2 silencing significantly decreased the activities of CAT, SOD, and T-AOC under normal culture, HS, and PB2 + HS conditions (T-AOC under HS condition, *p* < 0.05; others, *p* < 0.01). Additionally, compared to the si-Nrf2 + HS groups, 25 μM PB2 pretreatment failed to change CAT activity (*p* > 0.05), with SOD and T-AOC enhanced (*p* < 0.01).

**Figure 8:**
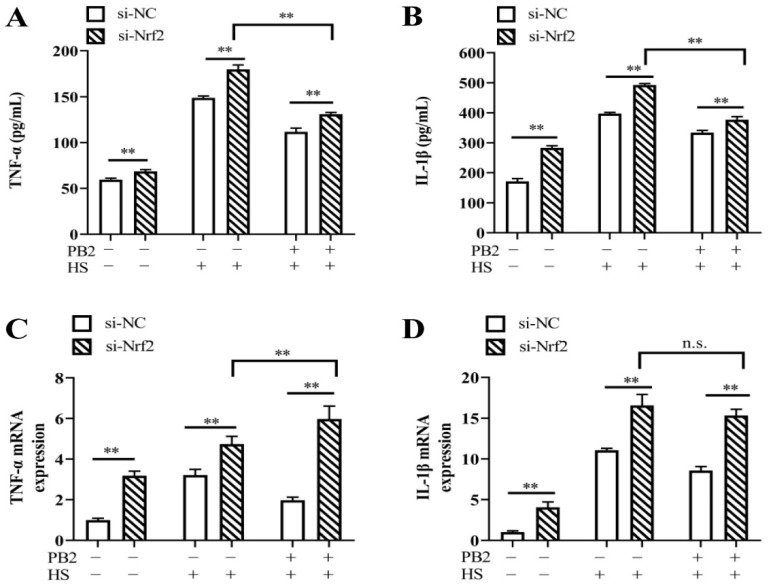
Effect of Nrf2 knockdown on release and gene expression of pro-inflammatory cytokines. MAC-T cells were transfected with si-Nrf2 or si-NC for 12 h, and then incubated with/without 25 μM PB2 for 24 h, followed by HS treatment for 3 h and recovery for 6 h. Then, the culture media were used to investigate the release of (**A**) TNF-α and (**B**) IL-1β, and cells were harvested to test (**C**) *TNF-α* and (**D**) *IL-1β* mRNA expression levels. *n* = 3. ** *p* < 0.01, n.s. *p* > 0.05.

To determine the role of the Nrf2 signaling pathway in mitigating the HS-induced inflammatory response, si-Nrf2 was again used to transfect MAC-T cells. The results in Figure 8 show that both the contents and mRNA expressions of *TNF-α* and *IL-1β* were increased after silencing Nrf2 in all conditions, including normal culture, HS treatment, and PB2 + HS treatment (*p* < 0.01). Compared to si-Nrf2 + HS-treated cells, 25 μM PB2 pretreatment under si-Nrf2 + HS condition significantly decreased the release of TNF-α and IL-1β (*p* < 0.01), while enhanced mRNA expressions of *TNF-α* (*p* < 0.01), with mRNA expressions of *IL-1β*, remained unchanged (*p* > 0.05). These results show that PB2 alleviates HS-induced oxidative stress and the production of pro-inflammatory cytokines, partly through the Nrf2 signaling pathway.

## 3. Discussion

HS refers to the non-specific physiological response of the body under the condition of high temperature, which is an important inducement for the body to produce oxidative stress [18]; it is also an important cause of mastitis in dairy cows, leading to a decline of milk production and quality [2]. PB2 is a polyphenolic flavonoid compound with anti-oxidative and anti-inflammatory effects [19,20]. This study shows that PB2 has a powerful antioxidant potential by inducing a variety of antioxidant genes and antioxidant enzymes to defensive oxidative stress in MAT-C cells caused by HS. Furthermore, PB2 exerts its antioxidant properties mainly by up-regulating the Nrf2 signaling pathway and reducing the release of pro-inflammatory factors. This study provides some evidence for the potential use of PB2 in dairy cows, in order to prevent and alleviate the adverse effects of HS.

Previous publications have shown different HS treatments on MAC-T cells, e.g., 42 °C for 12 h [10] and 42 °C for 8 h [21], as well as 42.5 °C for 1 h and recovery at 37 °C for 12 h [22], resulting in about 55%, 60%, and 70% of the cell viability, respectively. To establish an effective HS model in the study, MAC-T cells were exposed to 42 °C temperature for various time periods and recovered at 37 °C for 6 h to get suitable thermal treatment time. Then, 3 h of 42 °C treatment was used, and the cells were then recovered at 37 °C for different time periods to acquire appropriate recovery time. As treatment of HS at 42 °C for 3 h and recovery at 37 °C for 6 h was shown to be closest to reaching half of the cell viability, the above treatment was, therefore, used for the HS model in the present study. Furthermore, different concentrations of PB2 were applied on MAC-T cells. The results indicated that high doses of PB2 treatment for 24 h displayed a significant inhibitory effect on cell viability. Hence, low concentrations (5, 10, and 25 μM) of PB2 were used in the study to detect its protective effects under heat exposure. This study found that 5, 10, and 25 μM of PB2, effectively dose-dependently, alleviated the decline in cell viability induced by high temperature, which is consistent with the research results, which suggest that PB2 can ameliorate the reduction in neuronal cell viability caused by cypermethrin [23] and defense the viability loss of human umbilical vein endothelial cells under lipopolysaccharide (LPS)-stimulated condition [24].

Heat shock proteins (HSPs) are a main product of the stress response; they play an important role in maintaining cell survival and internal environmental stability [25]. Studies have revealed that HSP70 inhibits cell apoptosis [26], enhances cell thermal tolerance [27], resists oxidative stress [28], and participates in the biological functions of the body’s immunity and molecular chaperones. Among HSPs, HSP70 is the strongest heat-induced protein, and it protects cells during HS [29]. After HS exposure of mammary epithelial cells, the expression of HSP70 is highly increased, and methionine supplementation further enhances expressions of HSP70 compared with the HS-treated groups [30]. Similarly, our results show that PB2 did not alter HSP70 expression, while HS significantly induced the expression of HSP70 and PB2 pretreatment further increased HSP70 expression under HS condition. This result indicates that PB2 treatment on MAC-T cells under hyperthermia increased the heat tolerance of cells partly via upregulating HSP70 expression.

ROS are mainly generated in the mitochondria during aerobic metabolism. Under normal circumstances, the production and elimination of active oxygen in the body maintains a dynamic balance. The ROS level is an important indicator of the body’s oxidative status, and high levels of ROS result in protein oxidation, lipid peroxidation, and DNA damage, causing substantial damage to nearby tissues [31]. High temperature can stimulate the production of ROS, leading to the imbalance of the body’s antioxidant defense to generate oxidative stress [32]. On one hand, oxygen free radicals attack the polyunsaturated fatty acids in the biofilm to form lipid peroxide MDA, and the content of MDA can indirectly reflect the degree of cell damage [33]; on the other hand, CAT and SOD are primary enzymatic antioxidants that resist against ROS-induced oxidative damage. Specifically, SOD can catalyze superoxide free radicals to generate molecular oxygen and hydrogen peroxide, and CAT decomposes the hydrogen peroxide decomposed by SOD into water and oxygen. T-AOC reflects the total antioxidant capacity in the cell and can reflect the overall oxidative stress level in the body. HS can increase the ROS level and reduce CAT and SOD activity via oxidative inactivation [34]. Consistent with the results, in the present study, increased ROS and MDA levels, together with a significant decline of CAT, SOD, and T-AOC activity, were observed in MAC-T cells after heat exposure. Furthermore, as evidenced by all of the above markers, PB2 pretreatment dose-dependently alleviated the oxidative status induced by hyperthermia condition. Similarly, the anti-oxidative effects of PB2 have been also reported by previous studies. For example, PB2 can increase the activities of CAT and SOD in HepG2 cells and ameliorate oxidative stress induced by free fatty acids [35]. PB2 supplementation in the mice diet increases the activities of CAT and SOD and decreases MDA content in liver and/or plasma, resisting the oxidative stress caused by high fat diet [36] and carbon tetrachloride [36].

Oxidative stress and inflammation are intertwined and affect each other. Studies have shown that ROS is the major inducement of inflammation [37], while TNF-α is the main cytokine that induces inflammation; the increase of TNF-α promotes the production of ROS and up-regulates the oxidative stress response [38]. IL-1β is the main active form of interleukin-1 (IL-1), an important pro-inflammatory cytokine in the body that participates in the regulation of the immune response and inflammatory reaction process [39]. Previous studies report that PB2 inhibits the release of TNF-α and IL-1β in macrophages, thus alleviating the inflammatory response induced by LPS [40]. Consistently, our study reveals that high temperatures stimulate the up-regulation of the expressions and contents of TNF-α and IL-1β in MAT-C cells, and PB2 dose-dependently inhibits the increase of the pro-inflammatory cytokines induced by high temperature, thus indicating that PB2 protects MAT-C cells against HS-induced inflammation, which might be associated with the increased activities of antioxidant enzymes and reduced ROS content.

The Nrf2 signaling pathway is vital for cells to resist external stimuli and maintain the intracellular redox balance [41,42]. Intracellular oxidative stress activates Nrf2 translocation to the nucleus; then, Nrf2 binds to the antioxidant response element to regulate the expression of downstream antioxidant genes, including HO-1 and Txnrd1 [43]. In order to further explore the mechanism of the antioxidant effect of PB2, we tested the expressions of Nrf2, HO-1, and Txnrd1, as well as the translocation of Nrf2. Results show that HS promoted the expression and nuclear translocation of Nrf2, with the mRNA expressions of HO-1 and Txnrd1 increased. Furthermore, pretreatment with PB2 further stimulated Nrf2/HO-1 signaling activation under HS conditions, indicating PB2 may block the ubiquitination and degradation of Nrf2 and promote its nuclear translocation, thus leading to an increase in the expression of Nrf2 and its downstream antioxidant genes to protect cells [44]. Studies have shown that tert-Butylhydroquinone and resveratrol promote the nuclear translocation of Nrf2 and increase expressions of Nrf2, HO-1, and Txnrd1, thus protecting dairy mammary epithelial cells from oxidative stress induced by high temperatures and hydrogen peroxide [44,45], which are consistent with the present results. However, there are also research findings that show that choline protects bovine mammary epithelial cells from HS-induced oxidative stress by inhibiting the expression and nuclear translocation of Nrf2 [46]. Similarly, PB2 was reported to protect mice cerebral cortex neurons from cypermethrin-induced oxidative stress by reducing Nrf2 expression and nuclear translocation [24]. The reason for this difference might be related to the intensity of oxidative stress.

To further explore whether PB2 protected HS-induced MAC-T cells by regulating the Nrf2 signaling pathway, si-Nrf2 transfection targeting Nrf2 was then performed. The results show that, under HS and PB2 + HS conditions, Nrf2 knockdown significantly decreased cell viability, reduced gene expressions of HO-1 and Txnrd1, decreased activities of SOD, CAT, and T-AOC, and increased the contents of TNF-α and IL-1β. These results indicate that Nrf2 knockdown lost the key protective effect of PB2 on MAC-T cells. However, compared with the si-Nrf2 + HS-treated cells, PB2 pretreatment under si-Nrf2 + HS conditions weakened the protective effect of PB2 on cells, but still significantly improved cell survival rate, increased expressions of part antioxidant genes and enzymes, and decreased contents and expressions of part of the pro-inflammatory cytokines. These results indicate that the Nrf2 signaling pathway is a main defense mechanism of PB2 protection against HS-induced oxidative stress and inflammatory response; other signaling pathways might also be involved in this process. Consistent with the data, the addition of sirtuin 6 after si-Nrf2 transfection was also reported to increase HO-1 gene expression and still exert its protective effect on the LPS-induced apoptosis of human umbilical vein endothelial cells [47]. As PB2 has also been reported to alleviate cell oxidative stress through ERK and P38 MAPK signaling pathways [48], other molecular mechanism responsible for the PB2 protection effect on MAC-T cells need to be clarified in further studies.

## 4. Materials and Methods

### 4.1. Cell Culture and Treatment

MAC-T cells were cultured in DMEM/F12 (Hyclone, South Logan, UT, USA) media supplemented with 10% fetal bovine serum (FBS; Gibco, Carlsbad, CA, USA), 100 IU/mL penicillin, and 100 µg/mL streptomycin (Hyclone). The cells were incubated at 37 °C in a humidified incubator containing 5% CO_2_.

PB2 was purchased from MedChemExpress (MCE, Monmouth Junction, NJ, USA). To establish an in vitro HS cell model, cells were transferred from the 37 °C incubator to an adjacent 42 °C incubator for 0~12 h, and then returned at 37 °C incubator for 0~24 h. Various concentrations, ranging from 0 to 100 μM, of PB2 were added to the MAC-T cells for 12 or 24 h prior to HS treatment.

### 4.2. Cell Viability Assay

The cell viability evaluation was performed using the Cell Counting Kit-8 (CCK-8; Vazyme, Nanjing, China), according to the manufacturer’s protocol. Briefly, cells were seeded in 96-well culture plates (5 × 10^3^ cells per well) and incubated for 24 h, then applied with the indicated treatments. The cells were then treated with 10 μL of CCK-8 reagent for 4 h, followed by absorbance measurement at 450 nm with a microplate reader (Thermo Fisher Scientific, Waltham, MA, USA).

### 4.3. Antioxidant Capacity Measurement

Cells were seeded in 6-well culture plates and treated as described above. They were then harvested and lysed in ice-cold phosphate-buffered saline (PBS; Hyclone) by sonication, followed by centrifugation at 12,000× *g* for 10 min at 4 °C. The supernatant was taken for subsequent determination. The cellular catalase (CAT), superoxide dismutase (SOD), total antioxidant capacity (T-AOC), and malondialdehyde (MDA) were determined with corresponding assay kits (Nanjing Jiancheng Bioengineering Institute, Nanjing, China), according to the manufacturer’s instructions. The absorbance was measured with a microplate reader at 405, 450, 593, and 532 nm for CAT, SOD, T-AOC, and MDA, respectively. Cellular protein concentrations were measured using a bicinchoninic acid (BCA) protein assay kit (Vazyme) to normalize the above data.

### 4.4. Detection of ROS

The level of intracellular ROS was detected using a ROS assay kit (Beyotime, Shanghai, China). Briefly, cells were seeded on coverslips in culture dishes and treated. They were then washed by PBS and incubated with 10 μM 2′, 7′-dichlorodihydrofluorescein diacetate (DCFH-DA, green fluorescence) at 37 °C for 20 min, followed by washing thrice with PBS. Images of the cells were observed and captured using a Zeiss LSM700 confocal laser scanning microscope (Carl Zeiss, Oberkochen, Germany) at an excitation wavelength of 488 nm and emission wavelength of 525 nm. The above experiment was repeated three times. Image analysis was performed using ImageJ 1.8.0 software (National Institutes of Health, Bethesda, MD, USA).

### 4.5. Determination of IL-1β and TNF-α

Cells were seeded in 6-well culture plates and treated. Then, cell culture media were collected and centrifuged at 3000 r/min for 20 min at 4 °C. The supernatant was used for IL-1β and TNF-α measurements via corresponding enzyme-linked immunosorbent assay (ELISA) kits (Shanghai Enzyme-linked Biotechnology, Shanghai, China), according to instructions of the manufacturer. The amounts of IL-1β and TNFα were calculated from standard curves and expressed in total per milliliter (pg/mL).

### 4.6. Transient Transfection of Nrf2

The small interference RNA (siRNA)-targeting bovine *Nrf2* gene (si-Nrf2) and a negative control siRNA (si-NC) were synthesized by GenePharma. The sense sequence of si-Nrf2 is 5′-CAGUUGAGGACUUCAAUGAdTdT-3′, and the antisense sequence is 5′-UCAUUGAAGUCCUCAACUGdTdT-3′) [45]; the sense sequence of si-NC is 5′-UUCUCCGAACGUGUCACGUdTdT, and the antisense sequence is 5′- ACGUGACACGUUCGGAGAAdTdT-3′. Transfection was performed using lipofectamine 2000 (Invitrogen, Carlsbad, CA, USA), according to the manufacturer’s protocol. After 12 h of transfection, cells were treated as described above; the MAC-T cells were harvested 48 h after transfection to detect *Nrf2* mRNA levels.

### 4.7. RNA Isolation and Quantitative Real-Time PCR

Total RNA was extracted using TRIzol reagent (Invitrogen). The purity and quantity of RNA were checked using a Nanodrop 2000 spectrophotometer (Thermo Fisher Scientific). Next, 1.0 μg of total RNA was reverse-transcribed to obtain cDNA using the PrimeScript™ RT reagent kit (Takara, Tokyo, Japan ). The quantitative real-time PCR was performed using the StepOne Real-time PCR system (Applied Biosystems, Foster, CA, USA) and SYBR Premix Ex Taq™ (Takara). The PCR cycling conditions were as follows: 95 °C for 30 s, 40 cycles at 95 °C for 5 s, and 60 °C for 30 s. Primers were synthesized by Shanghai Sangon Biological Engineering Technology Services Co., Ltd (Shanghai, China). Gene expression quantification was performed using the 2^−ΔΔCt^ method. Glyceraldehyde-3-phosphate dehydrogenase (GAPDH) served as an endogenous control. The primer information is listed in Table 1.

### 4.8. Immunofluorescence Staining

Cells were plated on coverslips and treated as described above. For immunofluorescence staining, cells were fixed in 4% paraformaldehyde for 15 min, permeabilized with 0.5% Triton X-100 for 20 min, and blocked in 5% bovine serum albumin for 1 h. Cells were then incubated with the primary antibody against Nrf2 (1:200; Proteintech, Wuhan, China) overnight at 4 °C. After thrice washes with PBS, the cells were incubated with a secondary antibody for 50 min at room temperature, away from light. The nuclei of cells were stained with 4′,6-diamidino-2-phenylindole (DAPI; Beyotime, Shanghai, China). Images were obtained with the confocal laser scanning microscope (Carl Zeiss, Oberkochen, Germany).

### 4.9. Protein Extraction and Western Blotting

Proteins from each group of cells were extracted using radio immunoprecipitation assay (RIPA) lysis buffer (Beyotime). The protein concentrations were determined using a BCA protein assay kit (Vazyme). Equal amounts (10 μg) of proteins were separated by SDS-PAGE and transferred to polyvinylidene fluoride membranes (Millipore, Burlington, MA, USA). Membranes were blocked with 5% skim milk buffer for 1 h at room temperature, and then incubated with primary antibodies against HO-1, Nrf2, or GAPDH (1:1000; Proteintech) overnight at 4 °C. The membranes were washed three times, and then incubated with HRP-conjugated goat anti-rabbit secondary antibody (1:10000; Proteintech) for 1 h at room temperature. The protein bands were visualized with an enhanced chemiluminescence detection kit (Vazyme), and protein quantification was performed using ImageJ 1.8.0 software.

### 4.10. Statistical Analysis

All data were expressed as the mean ± standard error of the mean (SEM). Statistical analysis was carried out by SPSS 21.0 software (SPSS Inc., Chicago, IL, USA). Normal distribution was tested via the Shapiro–Wilk test. Comparisons among groups were performed with one-way analysis of variance (ANOVA), followed by Tukey’s test. Student’s *t*-test was used to assess differences between two groups. *p* < 0.05 and *p* < 0.01 indicated statistical significance and very significance, respectively. Each experiment was repeated at least three times.

## 5. Conclusions

**Figure 9:**
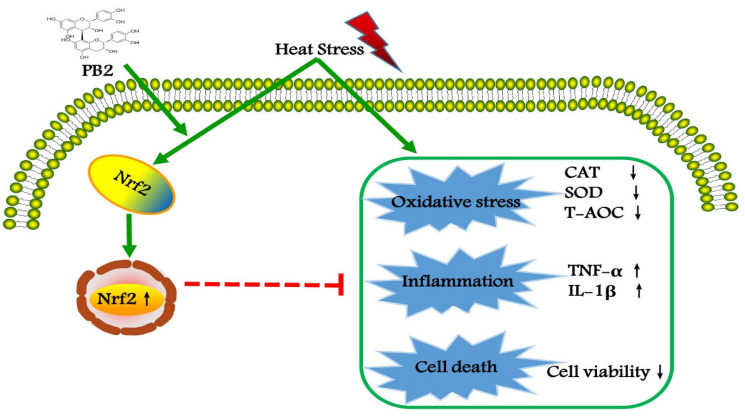
PB2 protects MAC-T cells against heat-induced oxidative stress and inflammatory response, predominantly via Nrf2 signaling pathway.

Taken together, HS stimulation induces oxidative stress and inflammation in MAC-T cells, resulting in significant impairment of cell viability. Preconditioning of PB2 can significantly improve the antioxidant defense ability of cells, partly through the Nrf2 signaling pathway, in order to alleviate the damage of HS to cells (Figure 9). These results provide functional evidence for PB2 as a new natural antioxidant to protect mammary epithelial cells from hyperthermia-induced damage. In vivo studies concerning PB2 supplementation in dairy cows in summer deserve further study.

## Figures and Tables

**Table 1:** Primer sequences for quantitative real-time PCR.

Gene	GenBank ID	Primer Sequences (5′ to 3′)	Product Size
*HSP70*	NM_203322.3	Forward: CAAGATCAGCGAGGCGGACAAG Reverse: ACACCTGCTCCAGCTCCTTCC	128 bp
*TNF-α*	NM_173966.3	Forward: CTGGCGGAGGAGGTGCTCTC Reverse: GGAGGAAGGAGAAGAGGCTGAGG	85 bp
*IL-1β*	NM_174093.1	Forward: ATGAAGAGCTGCATCCAACACCTG Reverse: ACCGACACCACCTGCCTGAAG	110 bp
*Nrf2*	NM_001011678.2	Forward: TCAGCCAGCACAACACATACCATC Reverse: ACGGGAATGTCTCTGCCAAAAGC	128 bp
*HO-1*	NM_001014912.1	Forward: CCGCTACCTGGGAGACCTGTC Reverse: ACTTGGTGGCACTGGCGATATTG	128 bp
*Txnrd1*	NM_174625.5	Forward: CGTGCCTTACATCTATGCCATCGG Reverse: TGGAGCCACCATACAGCCTCTG	110 bp
*GAPDH*	NM_001034034.2	Forward: TGCCCGTTCGACAGATAGCC Reverse: GCGACGATGTCCACTTTGCC	148 bp

## Data Availability

The data presented in this study are available on request from the corresponding author.
